# Efficacy of a Green Banana–Mixed Diet in the Management of Persistent Diarrhea: Protocol for an Open-Labeled, Randomized Controlled Trial

**DOI:** 10.2196/15759

**Published:** 2020-03-06

**Authors:** Monira Sarmin, Md Iqbal Hossain, Shoeb Bin Islam, Nur Haque Alam, Shafiqul Alam Sarker, M Munirul Islam, Mohammod Jobayer Chisti, S M Rafiqul Islam, Mustafa Mahfuz, Tahmeed Ahmed

**Affiliations:** 1 Nutrition and Clinical Services Division International Centre for Diarrhoeal Disease Research, Bangladesh Dhaka Hospital Dhaka Bangladesh

**Keywords:** persistent diarrhea, green banana, milk suji, rice suji, microbiota, metabolomics

## Abstract

**Background:**

Diarrhea is the second-leading cause of death in children under 5 years of age. In low- and middle-income countries, 3%-20% of acute diarrheal episodes become persistent diarrhea (PD) (ie, duration ≥14 days), which results in 36%-56% of all diarrheal deaths. In Bangladesh, PD causes >25% of diarrhea-related deaths. Commensal gut microbiota dysbiosis is increasingly recognized in the pathogenesis of PD. Hospital-based management of PD requires a hospital stay, which increases the risk of infection and hospital costs. The higher cost of treatment and high case-fatality rates reiterate PD as an important public health problem. At the International Centre for Diarrhoeal Disease Research, Bangladesh (icddr,b), for the last two decades, a consensus-based guideline has been followed for PD. Observation has revealed that green banana helps in the resolution of diarrhea. However, no larger prospective study has been conducted to evaluate the efficacy of green banana in the management of PD among children older than 6 months of age.

**Objective:**

Our objective is to assess the efficacy of full-strength rice suji (semolina) with and without green banana compared to three-quarter-strength rice suji in the management of PD in children aged 6-36 months at the Dhaka Hospital of the icddr,b.

**Methods:**

This open-labeled, randomized controlled study aims to enroll a total of 145 children with PD who have not been improving on a diet of milk suji. Children will be randomized into three different diet-specific groups: full-strength rice suji containing green banana, full-strength rice suji alone, and three-quarter-strength rice suji. The primary outcome is the percentage of children who recovered from diarrhea by day 5.

**Results:**

Recruitment and data collection began in December 2017 and were completed in November 2019. Results are expected by April 2020.

**Conclusions:**

This study is expected to provide insights into the incorporation of green banana into the dietary management of PD. This would be the first study to investigate the role of microbiota and metabolomics in the pathogenesis of PD.

**Trial Registration:**

ClinicalTrials.gov NCT03366740; https://clinicaltrials.gov/ct2/show/NCT03366740

**International Registered Report Identifier (IRRID):**

DERR1-10.2196/15759

## Introduction

Diarrhea, defined as the passage of loose or watery stool three or more times in a 24-hour period, is the second-leading cause of death in children under 5 years of age [1]. It accounted for 477,293 deaths among 5.4 million people globally and 7062 deaths among 99,608 Bangladeshi children under 5 years of age in 2017 [2]. Use of oral rehydration salt solution and zinc reduced the number of diarrheal deaths to 0.8%, especially from acute diarrhea. However, when diarrhea continues for 14 days or more, not including recurrent or chronic diarrhea as found with celiac disease, cystic fibrosis, or congenital disorders, it is known as persistent diarrhea (PD) [3]. In low- and middle-income countries, 3%-20% of acute diarrheal episodes turn into PD, which is responsible for 36%-56% of diarrhea-associated deaths [3-7]. In Bangladesh, PD accounted for more than 25% of all diarrhea-related deaths among children aged 1-4 years, of which 40% were malnourished [4]. A total of 60% of PD occurs before 6 months of age and 90% below 1 year of age [8]. Along with malnutrition, younger ages, lack of breastfeeding, infection, and inappropriate use of antibiotics are risk factors for PD [9-11]. Due to multifaceted etiology, proper diagnosis and treatment are often warranted for quick recovery from such episodes. In addition, the higher cost of treatment and high case fatality rates reiterate PD as an important public health problem [12]. Every year in Bangladesh, the Dhaka Hospital of the International Centre for Diarrhoeal Disease Research, Bangladesh (icddr,b)—the largest diarrheal hospital in the world—treats a number of children with PD; as well, PD cases peak during the summer [13]. In the management of PD, we follow the algorithm based on earlier studies from the Dhaka Hospital of icddr,b [14,15] and that suggested by the World Health Organization (WHO) [16]. It includes rehydration; control of infection, if present; algorithm-based dietary intervention; micronutrient supplementation; and management of associated complications.

**Figure 1:**
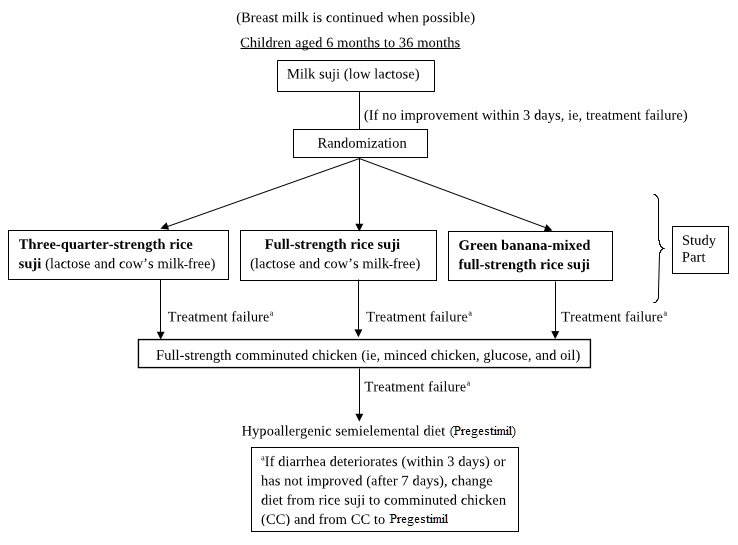
Flowchart for the proposed randomized controlled trial.

Algorithm-based dietary management increases the duration of hospital stay (see Figure 1). To reduce the osmotic burden to the gut, children are frequently given a diet three-quarters the strength of a regular diet. Though these diets are iso-osmolar, they provide suboptimal energy to the children. Prolonged diarrhea, diminished nutrient absorption [17], and low-calorie intake causes children to be malnourished. Consequently, there is an exaggerated risk of hospital-acquired infections, such as septicemia and pneumonia [18], with unwanted fatal outcomes, which pose a great burden to resource-poor settings. A group of icddr,b scientists have conducted studies to find a remedy for PD that includes green banana [19,20], guar gum [21], and probiotics [22].

Several studies demonstrated the beneficial effect of green banana (ie, whole green banana fruit, *Musa paradisiacal sapientum*) in the resolution of PD [19,20]. The antidiarrheal action of green banana is postulated to be mediated by its high content of amylase-resistant starch, which is not digested in the small intestine of humans [23,24]. On reaching the colon, this starch is fermented by resident bacteria into the short-chain fatty acids butyrate, propionate, and acetate [24]. In the colon, short-chain fatty acids stimulate salt and water absorption [25,26]; they also provide energy and induce a trophic effect on the colonic and the small-bowel mucosa [27].

The human body is home to trillions of microorganisms, primarily bacteria in the gut, which are generally referred to as the microbiota [28]. Commensal gut microbiota dysbiosis is increasingly recognized in the pathogenesis of PD [29]. Therefore, analysis of the commensal gut microbiota and adjusting the intestinal microbiota might be a promising method for the prevention or treatment of PD. In addition, there might be some proteins, factors, or host-pathogen interactions responsible for the continuation of diarrhea, which transform acute watery diarrhea into prolonged diarrhea and finally into PD [29]. Thus, we aimed to acquire knowledge about proteomics and metabolomics related to PD to explore the pathophysiological mechanisms of PD, which could lead to a targeted management strategy.

To address this background and knowledge gap, we have designed this randomized controlled clinical trial to include 6-36-month-old male and female children with PD. Our objective is to compare the efficacy of full-strength rice suji (semolina) containing green banana, full-strength rice suji without banana, and three-quarter-strength rice suji without banana in the resolution of PD in children. In addition, we will be able to evaluate the role of the gut microbiota and proteomics in the pathogenesis of PD.

## Methods

### Ethics and Research Approval

This study has received approval from the Institutional Review Board (IRB) at the icddr,b (approval No. 17075, version 3.0, dated October 22, 2017). Final data will be publicly disseminated regardless of the study results. A report containing the study results will be submitted for publication in an appropriate journal after completion of data collection and analysis. The study protocol follows the Standard Protocol Items: Recommendations for Interventional Trials (SPIRIT) guidance for protocol reporting [30] (see Tables 1 and 2).

All subjects will need to give written informed consent in accordance with the Declaration of Helsinki. The privacy, anonymity, and confidentiality of data and information identifying study participants will be strictly maintained. All medical information, description of treatment, and results from laboratory tests will be confidential and kept under lock and key; only the research staff will have access to this information. A quality assurance audit and inspection of this study may be conducted by the Ethical Committees of the IRB and will be independent of the study investigators and the sponsor; the quality assurance auditor will have access to all medical records, the investigator’s study-related files and correspondence, and the informed consent documentation relevant to this clinical study. The occurrence of any serious adverse event (eg, death) will be reported to the IRB within 24 hours of the event and their recommendations will be followed.

The study recruited patients and placed them into three different groups. An addendum to the protocol (version 4.0, dated May 5, 2018) has been approved by the IRB and includes a plan to collect stool and blood samples for microbiota and metabolomics analysis, respectively. The funder has not and will not have influence at any stage of the research, from study design to publication.

Traditionally, green banana is used as an antidiarrheal agent in the community. It is also used as a vegetable in Bangladesh, India, and other countries in Africa. Therefore, the IRB from the icddr,b did not direct us to form a data-monitoring committee.

**Table 1:** Standard Protocol Items: Recommendation for Interventional Trials (SPIRIT) schedule of enrollment, interventions, and assessments.

Protocol items		Study period					
		Screening (day -3 to -1)	Enrollment (day 0)	Assessment and allocation (day 0)	Study diets (days 2-7)	First follow-up (days 14±3)	Second follow-up (days 21±3)
**Enrollment**							
	Eligibility screen		x				
	Informed consent		x				
	Randomization and allocation			x			
**Interventions**							
	Different diets			x	x		
**Assessments**							
	Demographics	x					
	Comorbidities	x					
	Physical examinations	x			x	x	x
**Primary outcome**							
	Resolution of diarrhea (by day 5)				x		
**Secondary outcomes**							
	Resolution of diarrhea (by day 7)				x		
	Consistency of stool				x		
	Frequency of stool				x		
	Recovery time				x		
	Hospital-acquired infection				x		
	Relapse at first follow-up					x	
	Relapse at second follow-up						x
	Enteric pathogen detection by TaqMan assay^a^	x					
	Detection of gut microbiota by 16S rRNA sequencing^a^		x		x		
	Detection of proteomes and metabolomes from blood^a^		x		x		

^a^According to the addendum (Ethical Committee approval on May 5, 2018), at the end of the study, stored stool samples and blood samples will be processed for the desired testing.

**Table 2:** Standard Protocol Items: Recommendations for Interventional Trials (SPIRIT) 2013 checklist: recommended items are addressed in this clinical trial’s protocol and related documents.

Section or item, item no.			Description	Page no. where addressed
**Administrative information**				
	**Title**			
		1	Descriptive title identifying the study design, population, interventions, and, if applicable, trial acronym	1
	**Trial registration**			
		2a	Trial identifier and registry name; if not yet registered, name of intended registry (ie, ClinicalTrials.gov, ID: NCT03366740)	2
		2b	All items from the World Health Organization Trial Registration Data Set	N/A^a^
	**Protocol version**			
		3	Date and version identifier	4
	**Funding**			
		4	Sources and types of financial, material, and other support	20
	**Roles and responsibilities**			
		5a	Names, affiliations, and roles of protocol contributors	1
		5b	Name and contact information for the trial sponsor (ie, the icddr,b^b^)	N/A
		5c	Role of study sponsor and funders, if any, in study design; collection, management, analysis, and interpretation of data; writing of the report; and the decision to submit the report for publication, including whether they will have ultimate authority over any of these activities	5
		5d	Composition, roles, and responsibilities of the coordinating center, steering committee, end-point adjudication committee, data-management team, and other individuals or groups overseeing the trial, if applicable (see Item 21a for data-monitoring committee) (ie, Institutional Review Board and the icddr,b)	4, 5
**Introduction**				
	**Background and rationale**			
		6a	Description of research question and justification for undertaking the trial, including summary of relevant studies (published and unpublished) examining benefits and harms for each intervention	3, 4
		6b	Explanation for choice of comparators	3, 4
	**Objectives**			
		7	Specific objectives or hypotheses	18
	**Trial design**			
		8	Description of trial design, including type of trial (eg, parallel group, crossover, factorial, or single group), allocation ratio, and framework (eg, superiority, equivalence, noninferiority, and exploratory)	13, 14
**Methods: participants, interventions, and outcomes**				
	**Study setting**			
		9	Description of study settings (eg, community clinic and academic hospital) and list of countries where data will be collected; reference to where list of study sites can be obtained	12
	**Eligibility criteria**			
		10	Inclusion and exclusion criteria for participants; if applicable, eligibility criteria for study centers and individuals who will perform the interventions (eg, surgeons and psychotherapists)	13
	**Interventions**			
		11a	Interventions for each group with sufficient detail to allow for replication, including how and when they will be administered	13, 14
		11b	Criteria for discontinuing or modifying allocated interventions for a given trial participant (eg, drug dose change in response to harms, participant request, and improving or worsening disease)	17
		11c	Strategies to improve adherence to intervention protocols, and any procedures for monitoring adherence (eg, drug tablet return and laboratory tests)	N/A
		11d	Relevant concomitant care and interventions that are permitted or prohibited during the trial	17, 18
	**Outcomes**			
		12	Primary, secondary, and other outcomes, including the specific measurement variable (eg, systolic blood pressure), analysis metric (eg, change from baseline, final value, and time to event), method of aggregation (eg, median and proportion), and time point for each outcome; explanation of the clinical relevance of chosen efficacy and harm outcomes is strongly recommended	18
	**Participant timeline**			
		13	Time schedule of enrollment; interventions, including any run-ins and washouts; assessments; and visits for participants—a schematic diagram is highly recommended (see Table 1)	5, 6
	**Sample size**			
		14	Estimated number of participants needed to achieve study objectives and how it was determined, including clinical and statistical assumptions supporting any sample size calculations	19
	**Recruitment**			
		15	Strategies for achieving adequate participant enrollment to reach target sample size	N/A
**Methods: assignment of interventions (for controlled trials)**				
	**Allocation: sequence generation**			
		16a	Method of generating the allocation sequence (eg, computer-generated random numbers), and list of any factors for stratification; to reduce predictability of a random sequence, details of any planned restriction (eg, blocking) should be provided in a separate document that is unavailable to those who enroll participants or assign interventions (ie, block randomization)	18
	**Allocation: concealment mechanism**			
		16b	Mechanism of implementing the allocation sequence (eg, central telephone and sequentially numbered, opaque, sealed envelopes), describing any steps to conceal the sequence until interventions are assigned	18
	**Allocation: implementation**			
		16c	Study members who will generate the allocation sequence, will enroll participants, and will assign participants to interventions (ie, allocation sequence: senior scientist not related to the study; enrollment and assignment: principal investigator and study physician)	18
	**Allocation: blinding (masking)**			
		17a	Study members who will be blinded after assignment to interventions (eg, trial participants, care providers, outcome assessors, and data analysts), and how (ie, open-labeled trial)	18
		17b	If blinded, circumstances under which unblinding is permissible, and procedure for revealing a participant’s allocated intervention during the trial	N/A
**Methods: data collection, management, and analysis**				
	**Data collection methods**			
		18a	Plans for assessment and collection of outcome, baseline, and other trial data, including any related processes to promote data quality (eg, duplicate measurements and training of assessors) and a description of study instruments (eg, questionnaires and laboratory tests), along with their reliability and validity, if known; reference to where data collection forms can be found, if not in the protocol	15, 16
		18b	Plans to promote participant retention and complete follow-up, including list of any outcome data to be collected for participants who discontinue or deviate from intervention protocols	N/A
	**Data management**			
		19	Plans for data entry, coding, security, and storage, including any related processes to promote data quality (eg, double data entry and range checks for data values); reference to where details of data-management procedures can be found, if not in the protocol	19
	**Statistical methods**			
		20a	Statistical methods for analyzing primary and secondary outcomes; reference to where other details of the statistical analysis plan can be found, if not in the protocol	19
		20b	Methods for any additional analyses (eg, subgroup and adjusted analyses)	19
		20c	Definition of analysis population relating to protocol nonadherence (eg, as randomized analysis), and any statistical methods to handle missing data (eg, multiple imputation)	N/A
**Methods: monitoring**				
	**Data monitoring**			
		21a	Composition of data-monitoring committee, summary of its role and reporting structure, statement of whether it is independent from the sponsor and competing interests, and reference to where further details about its charter can be found, if not in the protocol; alternatively, an explanation of why a data-monitoring committee is not needed	5
		21b	Description of any interim analyses and stopping guidelines, including who will have access to these interim results and make the final decision to terminate the trial—no interim analyses in this trial	N/A
	**Harms**			
		22	Plans for collecting, assessing, reporting, and managing solicited and spontaneously reported adverse events and other unintended effects of trial interventions or trial conduct	5
	**Auditing**			
		23	Frequency and procedures for auditing trial conduct, if any, and whether the process will be independent from investigators and the sponsor	4
**Ethics and dissemination**				
	**Research ethics approval**			
		24	Plans for seeking research ethics committee (REC) and institutional review board (IRB) approval	4
	**Protocol amendments**			
		25	Plans for communicating important protocol modifications (eg, changes to eligibility criteria, outcomes, and analyses) to relevant parties (eg, investigators, RECs, IRBs, trial participants, trial registries, journals, and regulators)	5
	**Consent or assent**			
		26a	Study members who will obtain informed consent or assent from potential trial participants or authorized surrogates, and how (see Item 32) (ie, principal investigator and his or her representative)	N/A
		26b	Additional consent provisions for collection and use of participant data and biological specimens in ancillary studies, if applicable	N/A
	**Confidentiality**			
		27	How personal information about potential and enrolled participants will be collected, shared, and maintained in order to protect confidentiality before, during, and after the trial	4
	**Declaration of interests**			
		28	Financial and other competing interests for principal investigators for the overall trial and each study site	20
	**Access to data**			
		29	Statement of who will have access to the final trial dataset, and disclosure of contractual agreements that limit such access for investigators	4
	**Ancillary and posttrial care**			
		30	Provisions, if any, for ancillary and posttrial care and for compensation to those who suffer harm from trial participation	N/A
	**Dissemination policy**			
		31a	Plans for investigators and sponsor to communicate trial results to participants, health care professionals, the public, and other relevant groups (eg, via publication, reporting in results databases, and other data-sharing arrangements), including any publication restrictions	4
		31b	Authorship eligibility guidelines and any intended use of professional writers	N/A
		31c	Plans, if any, for granting public access to the full protocol, participant-level dataset, and statistical code	N/A
**Appendices**				
	**Informed consent materials**			
		32	Model consent form and other related documentation given to participants and authorized surrogates (see Multimedia Appendix 1)	N/A
	**Biological specimens**			
		33	Plans for collection, laboratory evaluation, and storage of biological specimens for genetic or molecular analysis in the current trial and for future use in ancillary studies, if applicable (see Table 1)	5, 6

^a^N/A: not applicable.

^b^icddr,b: International Centre for Diarrhoeal Disease Research, Bangladesh.

### Study Location

The study is being conducted at the Dhaka Hospital of the icddr,b, Dhaka, Bangladesh. This hospital provides care and treatment to over 166,624 patients annually of all ages and of both genders. Patients usually come with diarrheal illnesses and/or other associated problems, such as pneumonia, malnutrition, sepsis, and electrolyte abnormalities. In 2018, about 98,308 children under the age of 5 years were admitted. The majority of care seekers were from poor socioeconomic backgrounds and lived in urban and periurban Dhaka. Care is provided by a professional team, including junior and consultant physicians, nurses, counselors, and dietary workers in a multidisciplinary approach. There are different wards to treat patients with respiratory problems, diarrheal diseases, and malnutrition. The laboratory possesses well-equipped facilities capable of performing most of the clinical tests proposed in this study. For critically ill patients, there is an intensive care unit facility present within the hospital, equipped with necessary life support measures, including mechanical ventilators and syringe pumps for vasopressor support.

### Study Population

This study includes patients who meet the criteria below.

#### Inclusion Criteria

1. Children aged 6-36 months, having diarrhea for 14 days or more (up to 29 days), either at admission or developed at some point during their treatment period in the hospital.
2. Children able to take oral feeds at the time of randomization.

#### Exclusion Criteria

1. Children whose parents or caregivers do not provide consent.
2. Growth of Shigella, Salmonella, or Cholera in rectal swab culture.
3. Children having weight-for-length Z-scores or weight-for-height Z-scores of less than -5 SD or severe or generalized edema.
4. Children presenting with septic shock, convulsion, or any other illness that needs intensive care unit support during admission.
5. Birth defects, such as complex congenital heart diseases, cleft lip and cleft palate, Down syndrome, cerebral palsy, and others, that may themselves cause a digestive problem or failure to thrive.
6. Children diagnosed as having apparent or known tuberculosis, HIV, or chronic (>30 days) or organic diarrhea where the cause is known (eg, Crohn’s disease, ulcerative colitis, and celiac disease).

### Study Design

This is an open-labeled, randomized controlled clinical trial with three treatment arms (see Figure 1 and Table 3). Children 6-36 months of age admitted to the Dhaka Hospital of the icddr,b with PD or who developed PD during their treatment period and failed to respond with milk suji—a low-lactose formula made from milk powder and rice powder—have been screened and enrolled in this study. The participant enrollment period lasted 18 months.

### Randomization

The permuted block randomization technique was followed to select treatment arms for each child. The randomization procedure was planned and set up by a scientist from the icddr,b who was not involved in the data collection. The randomization list containing the subject IDs and the corresponding group allocation remained concealed. IDs were chronologically assigned to each new study participant. After randomization, opaque envelopes containing the names of the allocated diet groups were opened.

**Table 3:** Proposed dietary composition for this randomized controlled trial.

Ingredients per liter	Three-quarter-strength rice suji	Full-strength rice suji	Full-strength rice suji containing green banana
Green banana, g	N/A^a^	N/A	200
Glucose, g	30	30	25
Rice powder, g	40	60	50
Egg white, g	100	100	80
Soya bean oil, g	25	32	26
Sodium chloride, g	0.1	0.1	0.1
Magnesium chloride, g	0.5	0.5	0.5
Potassium chloride, g	1.0	1.0	1.0
Calcium carbonate, g	2.0	2.0	2.0
Energy, kcal/100 mL	57	70	70
Protein, g/100 mL	1.9	2.1	2.0
Osmolarity, mOsmol/L	296	298	<298
Protein energy ratio, %	13	12	11
Fat energy ratio, %	40	41	35

^a^N/A: not applicable.

### Collection of Baseline Information

All children with PD, either at admission or developed at some point during their treatment period, within the defined age group were screened for study eligibility criteria by the study physician. Parents or attending caregivers of those eligible children, depending on the inclusion and the exclusion criteria, were invited to provide their consent for enrollment of their children in the study. Parents and caregivers signed a written informed consent form (see Multimedia Appendix 1) and were provided with information about the study and its interventions, possible benefits and risks, and voluntary nature of participation, including the right to withdraw children at any time after the initial consent without providing any reason; after this, children were enrolled by the study physician. One copy of the signed consent document was given to the caregiver of each participant and another copy was kept for the study documentation. A pretested case record form was used to collect relevant medical history information, including nature and duration of illness and medication for current illness. The form was also used to collect information on sociodemographic characteristics, such as age, sex, religion, gestational age, parental age, parental education, parents’ occupations, drinking water source and sanitation, fuel use and smoking history, monthly family income, number of siblings, and number of rooms in the home. Information was also collected about each child’s feeding practice, such as their history of breastfeeding, formula feedings, or other complementary feedings, as well as immunization status and each child’s past history of pneumonia and diarrhea.

Data on clinical characteristics of participants was also collected. Clinical examination measurements recorded by the study physician included pulse and respiratory rate, axillary temperature, anthropometric measurements (ie, height, weight, mid-upper-arm circumference, weight-for-age Z-score, weight-for-length Z-score, and weight-for-height Z-score), chest auscultation, oxygen saturation, presence or absence of chest-wall indrawing, cyanosis, and mental status (ie, normal, irritable, or lethargic). Weight was measured using an electronic weighing scale with a precision of 0.1 kg; height/length was measured using a locally made length board with a precision of 0.5 cm by a trained and experienced nurse from the Dhaka Hospital of the icddr,b. Fever was defined when the axillary temperature was 38°C or greater. Respiratory rate was counted for a full 60 seconds by exposing the trunk when the child was awake and calm; the presence of lower-chest-wall indrawing was noted at the same time. Frequency and consistency of stool were monitored by either the study physician or a health worker every 8 hours up to the resolution of the diarrhea.

At discharge, caregivers were asked to come back with the children for a minimum of two, weekly follow-up visits.

### Laboratory Tests

All laboratory tests were carried out according to the management outline of PD based on the previous studies [31-34], which include the following tests:

1. Stool for routine microscopic examination and culture for Vibrio, Salmonella, Shigella, and Campylobacter jejuni from rectal swab culture.
2. Total and differential blood count.
3. Chest x-ray of anteroposterior view for management of pneumonia.
4. Serum electrolyte and creatinine if there is any clinical evidence of electrolyte imbalance or renal insufficiency.
5. Blood culture for suspected septicemia, typhoid fever, prolonged febrile illness, or hospital-acquired infection.
6. A rapid diagnostic test of stool by enzyme-linked immunosorbent assay (ELISA) for the diagnosis of Cryptosporidium spp and Giardia in selected cases, where the response is delayed or there is strong clinical suspicion.

With the aim to evaluate gut microbiota and proteomics in PD, additional investigations are planned as follows:

1. For gut microbiota analysis, fecal samples (2 g each) were collected from every child over the course of the study as follows: (1) on enrollment day, (2) at any time a diet was changed, and (3) at the time of discharge. Different types of microbiota of diverse groups (eg, Bacteroides, Prevotella, and Ruminococcus) will be tested by 16S rRNA sequencing.
2. For the TaqMan assay to detect other enteropathogens causing PD, a fecal sample (2 g) on screening day was collected.

For proteomic and metabolomic assays, two plasma samples (150 µL each) were collected from 50 children over the course of the study as follows: (1) on enrollment day and (2) at the time of discharge from the study; samples are to be analyzed and tested by a collaborative institute (to be decided).

### Clinical Management

Children with PD were admitted to the long-stay unit of the Dhaka Hospital of the icddr,b. Initial routine investigations were completed to identify the etiology of diarrhea by performing stool routine microscopic examinations and rectal swabs; if stool routine examinations were suggestive of invasive diarrhea, an appropriate antibiotic was provided according to hospital protocol. During this period, milk suji, a low-lactose milk and rice flour-based diet containing ~67 kcal and 1.3 g protein per 100 mL, was given as a routine diet. If the PD resolved with milk suji, the child was discharged with health advice. On day 4 (ie, 3 days after milk suji was given), if diarrhea did not resolve, the child was enrolled in the study and randomization was performed. The child received one of the three diets: full-strength rice suji containing green banana, full-strength rice suji alone, or three-quarter-strength rice suji alone. Children on all three diets were followed for 7 days. If there was deterioration of diarrhea (ie, either increased frequency or watery consistency) for 3 days or if the condition remained static for up to 7 days, the child’s status was declared as *treatment failure*. Children whose treatment failed received dietary intervention as per the standard management of diarrhea at the Dhaka Hospital of the icddr,b (see Figure 1).

### Volume of Diet

In this age group, we usually provide oral feeding equivalent to 60-85 mL/kg/day (12 feeds/24 hours). If breast milk was insufficient or the child was formula fed, the child received 120 mL/kg/day (12 feeds/24 hours). For a severely malnourished child who often did not get sufficient breast milk, the diet volume was 120 mL/kg/day (12 feeds/24 hours). Later, if a child demanded more and diarrhea had not worsened, the amount they were fed was increased to 144 mL/kg/day. The consultant physician made the final decision about the dietary volume, depending on the clinical condition of each patient. The volume offered and actual intake were properly recorded.

### Follow-Up After Discharge

Children who were fed full-strength rice suji, with or without green banana, or three-quarter-strength rice suji were discharged and were required to follow up for 2 weeks. At the end of 14 days, they returned to the hospital and if they remained diarrhea free, the diet was switched back to milk suji and caregivers were advised to introduce other family diet items gradually. Children with severe acute malnutrition and severe pneumonia received treatment according to the hospital’s standard management protocol [35,36] and WHO guidelines [32], respectively. Last but not the least, it is important to mention that caregivers who were the mothers of the children were encouraged to continue breastfeeding along with providing their children with a specific diet. The food in the study was prepared and provided by health workers under the close supervision of a qualified dietician; the diets were formulated based on locally available, culturally acceptable, affordable foods, quite similar to the WHO’s recommendations [3,14,16].

### Outcome Measures

#### Primary Outcome Variable

The primary outcome variable is the percentage of children who recovered from diarrhea by day 5 after being on the study diets.

#### Secondary Outcome Variables

There were eight secondary outcome variables, as follows: (1) the percentage of children who recovered from diarrhea by day 7, (2) the consistency of stool on different days, (3) the frequency of stool on different days, (4) the outcome after being on the study diets for 1 week (eg, recovery time), (5) the number of hospital-acquired infections, (6) the rate of relapse within 14 days of follow-up, (7) the detection of enteric pathogens via the TaqMan assay and gut microbiota via 16S rRNA sequencing, and (8) the detection of proteomes and metabolomes related to PD.

### Sample Size

Rabbani et al [20] conducted a study where they enrolled 5-12-month-old children; they found that their recovery rates from PD by day 4 on the green banana diet and rice suji was 78% and 23%, respectively. However, studies by Islam et al [13] and Mahfuz et al [37] found that a higher percentage of children recovered from PD after being given rice suji. There has been no study conducted where diets of rice suji with or without green banana were given to children 6-36 months of age. Considering these facts, we assumed that in 6-36-month-old children, the rate of recovery from diarrhea by day 5 in either of the intervention diet groups (ie, full-strength rice suji with or without green banana) would be 90% and that the rate of recovery in the control diet group (ie, three-quarter-strength rice suji) would be 65%.

With 80% power and a 5% type I error, and considering three treatment arms, we needed 40 children in each arm. If we enrolled 45 children in each arm, that would accommodate up to an 11% rate of attrition. Therefore, the total target sample size was 135 children (ie, 45 children × 3 arms).

### Statistical Analysis

Data has been entered into a personal computer and will be analyzed using SPSS Statistics for Windows, version 20.0 (IBM Corp). Statistical analyses will include descriptive as well as analytical methods. We will compare different characteristics (eg, age, gender, diarrhea and its duration, stool consistency, presence or absence of blood, fast breathing, lower-chest-wall indrawing, and fever). We will also evaluate the PD outcome as *improved*, *death in hospital*, *relapse*, or *death during follow-up*. Categorical variables will be compared using the chi-square test. When the variables of interest are continuous and parametric, the statistical significance of group mean comparisons will be evaluated by analysis of variance (ANOVA). When the main outcome measures are continuous nonparametric variables, the statistical significance of differences will be determined using the Kruskal-Wallis test. The post hoc test will be done accordingly. A probability of less than .05 will be considered statistically significant. Strength of association will be determined by calculating relative risk and 95% CI. Our primary analysis is an intention-to-treat analysis comparing the groups. Survival analysis will also be carried out. Finally, regression analyses will be performed to reach more definitive conclusions.

## Results

Recruitment and data collection began in December 2017 and were completed in November 2019. Results are expected by April 2020.

## Discussion

The purpose of this clinical research is to improve the existing standard dietary treatment to manage PD. PD incurs a great amount of morbidity and has accounted for about 36%-56% of diarrhea-related mortality in low- and middle-income countries [3-7]. Current algorithm-based management was developed several years ago; different countries adopted these guidelines with modifications depending on their local and cultural backgrounds. This study will focus on whether a low-calorie, low-osmolarity, or green banana diet works best for the early resolution of diarrhea. The data gathered from this study will hopefully be of great interest to scientists, who may seek to modify the management strategy of PD or to pursue new elements in PD where proteomics- and metabolomics-based studies might provide more answers.

In conclusion, this study is expected to provide useful insights into the efficacy of green banana in the management of PD in children aged 6-36 months. If this diet results in improved outcomes in this setting, then we can assume that it would also be beneficial in other similar health care facilities. We will demonstrate whether this simple yet practical solution for PD works or not.

## Appendix

Multimedia Appendix 1 Participant consent form.

Multimedia Appendix 2 Previous peer review reports from icddr,b.

## Abbreviations

ANOVA: analysis of variance
ELISA: enzyme-linked immunosorbent assay
icddr,b: International Centre for Diarrhoeal Disease Research, Bangladesh
IRB: Institutional Review Board
PD: persistent diarrhea
SPIRIT: Standard Protocol Items: Recommendations for Interventional Trials
WHO: World Health Organization
