# Supplementary material for: Efficacy of a Green Banana–Mixed Diet in the Management of Persistent Diarrhea: Protocol for an Open-Labeled, Randomized Controlled Trial
Source: JMIR Res Protoc. 2020 Mar 6;9(3):e15759. doi: 10.2196/15759 (PMC7154927; doi:10.2196/15759)
Supplement: Multimedia Appendix 1 [file resprot_v9i3e15759_app1.doc]

# consent Form (English)

| Protocol No. PR-17075 | Version No. 2.2 | Date: 04-04-2018 |
| --- | --- | --- |

Protocol Title: **Efficacy of green banana mixed full strength rice suji, and full strength rice suji alone compared to 3/4th strength rice suji in the management of persistent diarrhea (PD) in children aged >6 months to 36 months: an open-labeled randomized controlled clinical trial**

Investigator’s name: Dr. Monira Sarmin

Organization: icddr,b

**Purpose of the research**

**Background** (brief introduction of the issue and the need for/ importance of the research)

Diarrhea, defined as passage of loose or watery stool 3 times or more in a 24 hour period, is the 2^nd^ leading cause of death in under-five children. Use of ORS and zinc reduce the number of diarrheal deaths. However, when diarrhea continued for 14 days or more it is known as Persistent Diarrhea (PD). PD has been responsible for 32-62% of diarrhea associated deaths of young children in low and middle-income countries, and in Bangladesh PD accounted for more than 25% of diarrheal deaths among children aged 1-4 years.

**Why invited to participate in the study?**

We are trying to test the efficacy of cooked green banana when mixed with a full strength rice suji in the management of PD with the hope that it might help in early resolution of PD. To document this, we need to conduct the study on children above 6 months of age having PD. As your child has PD, this child is the right one to participate in this study and this is why we are inviting your child to help us by participating in this study.

**Methods and procedures [What is expected from the participants of the research study?]**

If you agree to our proposal of including your child in the study, you might expect the following:

- Your child will remain in the Longer Stay Unit (LSU) of this hospital, and we will provide appropriate care and treatment, according to the standard guideline of the hospital until the study ends.
- Your child will be monitored for 72 hours before enrollment in the study. At screening, one stool sample will be collected and stored for investigation, other investigation will be carried out according to hospital`s standard guideline. During this 72 hours period, the child will get standard diet milk suji as the norm of the hospital.
- If, PD resolved within this period, the child will be discharged. If still diarrhea continued, then the child will be enrolled in the study.
- After enrollment in the study, we would ask you some questions related to the current illness and health condition before this illness and perform thorough physical examinations on each day until your child remain(s) in the study to assess the progress of illness (improvement or deterioration).
- The child will be randomized and will get either of the three diets-
  - 1. Green banana mixed full strength rise suji,
    2. Full strength rice suji, or
    3. 3/4th strength rice suji.
- By any of these diets, your child will be followed for 7 days. If there is deterioration of diarrhea (either increased frequency or watery consistency) for 3 days or condition remains static up to 7 days the child will be declared as treatment failure. Because this study duration will be for 7 days, after these one week of management, if the child needs further hospital management, s/he will receive standard management of the Dhaka hospital of icddr,b.
- With the study diet, if the child`s PD resolute, the child will be followed up for the next 24 hours and will be planned for discharge. You will be taught how to prepare the study diet and the child will be discharged with common health related advices. You will also be advised to come weekly for follow up for coming two weeks

.

- It is prudent to mention, that this algorithm will be followed as a standard of care at the Dhaka Hospital of icddr,b for the management of PD during the study period. However, an expert consultant with their year-long experience in managing PD can take steps not mentioned in the guideline when thinks appropriate with proper documentation of the actions.
- For this research purpose, we will collect fecal samples (2g each) [on screening (for all eligible patients), on enrollment, discharge, and any time diet is changed after enrollment (for enrolled patients only)] and venous blood specimen by syringe two times during treatment: on enrollment and discharge (for enrolled patients only). Each blood specimen will be the same amount as about 1.0 ml. Stool specimen and the blood sample will be stored at icddr,b laboratory for future study. We assure you that the specimens will be used for no other purpose.

We also collect dietary intake data of the child through a questionnaire for last 24 hours at the time of stool collection.

However, we will record the results of other investigations done as a routine practice by the treating physicians for appropriate care of your child suffering from persistent diarrhea.

The management of your child and her/his discharge will not be influenced by the participation of your child in our study.

Follow up plan after discharge

- At the first and second follow up, history will be taken about diarrhea and its relapse if any and other complaints; vital signs and anthropometry will be recorded.
- At the end of 14 days, in 2^nd^ follow up if the child remained diarrhea free, the diet will be switched back to milk suiji and you will be advised to introduce other family diets gradually.
- Parents or caregivers of SAM children will be requested to stay at the nutritional rehabilitation unit (NRU) for the nutritional rehabilitation phase management of SAM. From NRU, the child will be discharged upon fulfilling the NRU criteria.

**Risk and benefits**

There is no major risk of your child’s participation in this study. With the exception of specimen collection, the treatment and care of patients will not differ from what would be routinely done at this hospital. No increased risk above the minimal level for stool and blood sample collection. While collection of blood your child will feel mild pain like ant bite during insertion of needle. There is small chance of temporary discoloration of surrounding skin. Other than this it will not cause any other harm to your child. We will use sterile, disposable syringes and needles & expert physician/nurse will draw blood. The results of this study are likely to benefit the society in the future by improving our knowledge and developing better treatment for persistent diarrhea leading to better outcomes.

**Privacy, anonymity, and confidentiality**

We do hereby affirm that privacy, anonymity and confidentiality of data/information identifying you/your patient will strictly be maintained. We would keep all medical information, description of treatment, and results of the laboratory tests collected from treating physicians confidential, under lock and key, and none other than our research staff will have an access to these information. We want to inform you that samples related to the study will be sent to USA for analysis.

**Future use of information**

In case of future use of the information collected from the study, anonymous or abstracted information and data would be supplied to other researchers, which should not conflict with or violate the maintenance of privacy, anonymity, and confidentiality of information identifying participants in any way.

**Right not to participate and withdraw**

Participation of your child in this study is voluntary. Refusal to take part in the study will involve no breach in the continuity of care and your child will receive the standard treatment of this hospital. You have a right to withdraw your child at any time from the study and you also have right to refuse to answer any question. Even if you withdraw your child from the study, your child will receive the usual good care in this hospital.

**Principle of compensation**

Treatment at this hospital is free for all patients, and your child will not be an exception. Similarly, we will not pay money for participating in our study.

**Answering your questions/ Contact persons**

We will happily provide you further information about the study, if any, now or at a later time. You may communicate with the principal investigators of the study at the contact address given below. We will answer your question related to your child’s medical condition, treatment. However, we would like to inform you that some tests will be performed at the end of the study, and thus their results would be available only then.

**Declaration by the parent/guardian:** The investigator(s) of the Research Project have explained to me the purpose, procedure, risks and benefits of this study; my right as the study participant; and on the confidential handling of the records of my child, including personal information. Based on these, I agree to my child’s participation in this study. I understand that I may end participation of my child in the study at any time without showing any cause, which will not change the care and treatment of my child at this hospital. I agree with the following study procedure:

Asking questions: Yes No Physical examination: Yes No

Access to medical record: Yes No

If you agree to our proposal of enrolling you/your patient in our study, please indicate that by putting your signature or your left thumb impression of the specified space below

Thank you for your cooperation

_______________________________________ ____________________

Signature or left thumb impression of Date

Parent/ Guardian/ Attendant/care giver

_______________________________________ ____________________

Signature or left thumb impression of the witness Date

_______________________________________ ___________________

Signature of the PI or his/her representative Date

# (NOTE: In case of representatives of the PI, she/he shall put her/his full name and designation and then sign)

# (Name and contact phone of IRB Secretariat, RA, M. A. Salam Khan, Phone No: 9886498 or PABX 8860523-32 Extension. 3206; Principle investigator: Dr. Monira Sarmin, Phone: +880-2-886-0523-32 ext 2816).
