# Supplementary material for: Efficacy of a Green Banana–Mixed Diet in the Management of Persistent Diarrhea: Protocol for an Open-Labeled, Randomized Controlled Trial
Source: JMIR Res Protoc. 2020 Mar 6;9(3):e15759. doi: 10.2196/15759 (PMC7154927; doi:10.2196/15759)
Supplement: Multimedia Appendix 2 [file resprot_v9i3e15759_app2.pdf]

Referee's Opinions

Referee 1:
Title: Efficacy of green banana mixed full strength rice suji, and full strength rice suji compared to $3/4^{th}$ strength rice suji in the management of persistent diarrhea (PD) in children aged > 6 months to 60 months: an open labeled randomized controlled clinical trial

Summary of Referee's Opinions: Please see the following table to evaluate the various aspects of the proposal by checking the appropriate boxes. Your detailed comments are sought on a separate, attached page.

	**Rank Score**		
	High	Medium	Low
Quality of project			√
Adequacy of project design			√
Suitability of methodology			√
Feasibility within time period		√	
Appropriateness of budget		√	
Potential value of field of knowledge			√

CONCLUSIONS

I support the application:

a) without qualification ☐

b) with qualification

- on technical grounds √ ☐
- on level of financial support ☐

I do not support the application ☐

Name of Referee: C A Kawser

Signature:

Date: 22nd July, 2017

Position: Professor

Institution: BSMMU

Response to comment from Prof C A Kawser on the research protocol

Sir, Thank you so much for your explicit review of the protocol.
We have discussed among the investigators. Here we have added our response to individual comment after each comment *italic* font and also revised the protocol accordingly.

RRC:

- Not seen it properly in the section "**icddr,b Strategic Priority/ Initiative (SP 2015-8)"**
- *Thank you for the suggestion. We have changed the strategic priority accordingly. (page no 1)*

- Is it necessary to link the identity of the individual?
- *During data collection, there will be an identifier link that will help to retrieve any missing data until analysis. But all the data will be kept confidential and the report/manuscript/article will be published without the name or any sorts of identification of the children studied*

- But may request to tick 'yes' for 'internal publication' as well as 'sharing with' GOB and NGO
- *"Yes" is marked to the options as per your suggestion*

Protocol itself:

- It seems there are three 'intervention' arms without a control. In the background section, protocol suggests 'milk-suji' followed by 'rice-suji' (no mention of strength) as a dietary intervention for PD but that intervention is not taken as controls.
- *At icddr,b,conventionally ¾ th strength rice suji is used for the management of PD in > 6 months to 60 months old children who did not improve with milk suji. And that is the control diet.*

- ¾ strength rice-suji has calorie conc. of 57 Kcal/100ml which is quite low and needs justification, with reference, of its use.
- *We agree with your comment. However, the children usually suffer from osmotic diarrhea in this stage. Diet with sufficient caloric density is often unsuccessful. Considering these facts we are proposing to raise the calorie by using full strength diet in the trial. This is described in details on page 20, paragraph 2 in the revised protocol.*

- The main objective is 'efficacy' of green banana in full-strength rice-suji compared to other two arms. But what are the 'outcome' variables that will be taken as 'efficacy'?
- *Outcome variable "Duration of diarrhea" will be taken as efficacy*

- There are three other secondary objectives. Is it necessary to have the first one, is it not well described! Regarding the third, please mention specific outcome variables to be compared.
  We appreciate your suggestion and these are addressed in the objective/outcome section of the revised protocol (page 16).

- There was a publication by G H Rabbani on the same topic, but ‘background’ section did not elaborate on it to justify the ‘repetition’ of the study. In sample size calculation the same study was referred while selecting the sample size.
- *The study conducted by Rabbani et al was a good study where the study enrolled only male children who were well nourished without any comorbid condition and also involved a small sample size. According to your suggestion, it was discussed in the background section.*

- In background section it was claimed that existing dietary intervention causing good outcome in 95% of cases. Then what are the changes in outcome is expected!!!?? If expected then that should be depicted in sample size by reducing ‘bad outcome’ of 5% to lower value. In that case sample size calculation will be totally different and possibly much larger than what is contemplated.
- *The study described 95% success rate was actually the cumulative cure rate even after using pregestimil and was a retrospective descriptive study. We think the precise information on the percentage of the >6 months to 60 months old children improved from start to end of using 3/4th strength rice suji is difficult to get from that study. So we have now not using that information in the revised protocol.*

- At ‘background’ few points were termed as ‘prognostic’ indicator, but are they not considered as ‘risk factor’?
- *Yes, these are risk factors. These are changed according to your suggestion*

- A long section was described in ‘first person’, but needs to be expressed in third person. The section described the existing management protocol but did not identify the lacunae or problem question/s that drives the present study.
- *According to your suggestion, the entire section was revised with a description of problems for which the study was planned.*

- Fig-3 is not properly shown.
- *It was reproduced from an article, as it was, it lacks some information. We have planned not to use it and deleted in the revised protocol.*

- Why a second exclusion criterion is kept? Other co-infections are to be attended. What is the chance of positivity for cholera in such cases?
- *Around 10% of patients with persistent diarrhea had growth of organisms in their stool and required antibiotics. If in one arm, more patients have growth than other groups, then it may produce problems during analysis.*

- Why -5 SD is taken an exclusion criteria, not -3 or -4? Needs justification.
- *We have plans to include children with PD and SAM. However, if a child has extreme malnutrition (Z score -5 or less), s/he needs closer monitoring, feeding through naso-gastric tube, so excluded from the study.*

- Please justify the volume of feed to be provided in the study. If a child is not breastfed, all the children will get <70 Kcal/kg/d. For partially breastfed, this amount will be much lower. It needs careful reconsideration/justification.

- *Thank you for expressing your concern about the calorie. Catch up growth is not usually the target during the dietary-management of the children suffering from PD and failed on milk suji diet. Of these children higher calories by offering higher volume often result in worsening of diarrhea. ~ 70 Kcal/kg will at least maintain resting metabolic rate of the children. If child demand more and diarrhea does not worsen amount of feed is increased to 12 ml/kg/feed every 2 hours/24 hours*

- On p-29 of the protocol, it is stated that children will be 'discharged' once bowel improves. Does it mean the child will be kept on <70 Kcal/kg/d diet?
- *Usually, children without malnutrition were discharged and the caregivers are advised to continue rice suji diet for 14 days. Within this period, they were followed up for 2 times and at the end of 14 days, they return to the hospital and if improved the diet is switched back to milk suiji and gradually other family food is introduced. Malnourished children were counseled to stay at NRU.*

- There should be number of operational definitions that will address the 'outcome' variables for 'outcome efficacy' (will it be only duration of diarrhea? Why not adding start of weight gain, frequency of hospital acquired infection, etc.) for the main objective. There should be another 'outcome' variables for other 'secondary' objectives.
- *To evaluate the efficacy (our primary objective), duration of diarrhea is the primary outcome variable and we have calculated the sample size for this outcome. For other variables, we may not be able to get a sufficient sample within the limited time period available for this study. Although those are important to see, we have considered them as secondary outcome variables and rewritten accordingly in the revised protocol.*

- While determining the sample size, ref 38 was used. But this reference used green banana with rice-suji. Then what difference is made in the protocol that may cause a 30% reduction in diarrhea duration? This needs to be explicitly described.
- *We have calculated the sample in a conservative way (so assumed 25% reduction in days), as we are also planning to enroll children with co-morbidities and/or severe and moderate undernutrition. If we assume 30% reduction then lesser number would be enrolled.*

- In the 'analysis' section, three categories made as improved, partial improvement and death. How the first two to be defined?
- *Sir, as the terms are not clear, We have defined the PD outcome as improved, death at hospital, relapse or death during follow up (page 31).*

Referee 2:

Title: Efficacy of green banana mixed full strength rice suji, and full strength rice suji compared to 3/4th strength rice suji in the management of persistent diarrhea (PD) in children aged >6 months to 60 months: an open-labeled randomized controlled clinical trial

Summary of Referee's Opinions: Please see the following table to evaluate the various aspects of the proposal by checking the appropriate boxes. Your detailed comments are sought on a separate, attached page.

	**Rank Score**		
	High	Medium	Low
Quality of project		√	
Adequacy of project design	√		
Suitability of methodology	√		
Feasibility within time period		√	
Appropriateness of budget		√	
Potential value of field of knowledge		√	

CONCLUSIONS

I support the application:

a) without qualification √ ☐

b) with qualification

- on technical grounds ☐
- on level of financial support ☐

I do not support the application ☐

Name of Referee: Prof. Sayed Shafi Ahmed Muaz

Signature: Date: 013.08.2017

Position: Professor and Head, Dept. of Pediatric Gastroenterology, Hepatology & Nutrition

Institution: Bangladesh Institute of Child Health and Dhaka Shishu (Children) Hospital

Response to comment from Prof Muaz on the research protocol

Sir, Thank you so much for your review of the protocol.
We have discussed among the investigators. Here we have added our response to individual comment after each comment *italic* font and also revised the protocol accordingly.
<u>RRC:</u>

Reviewer:

- I have thoroughly reviewed the project proposal and I have not found anything objectionable for ethical clearance. But will there be any problem of osmolality regarding use of green banana mixed full strength rice suji in PD where the osmolality is not known in the given diet.

- *Thank you, sir, for your concern regarding osmolality of the study diet when green banana will be added to rice suji. The osmolality of GB mixed full strength rice suji will be less than that of full strength rice suji. At this moment, our nutritional biochemistry lab is not doing the investigation. The exact value is under experiment and will be provided later.*
